# Supplementary material for: The Relationship between Serum miRNAs and Early Mortality in Multiple Myeloma Patients Treated with Bortezomib-Based Regimens
Source: Int J Mol Sci. 2023 Feb 2;24(3):2938. doi: 10.3390/ijms24032938 (PMC9917942; doi:10.3390/ijms24032938)
Supplement: Supplementary file 1 [file ijms-24-02938-s001.zip › Supplementary Table S1.pdf]

miRBase ID	GeneGlobe ID	Mature miRNA sequence
**miRNAs used for data normalization**		
hsa-miR-152-3p	YP00204294	UCAGUGCAUGACAGAACUUGG
hsa-miR-23b-3p	YP02119314	AUCACAUUGCCAGGGAUUACCAC
**selected miRNAs used for the expression analysis**		
hsa-miR-122-5p	YP00205664	UGGAGUGUGACAAUGGUGUUUG
hsa-miR-1224-3p	YP00204045	CCCCACCUCCUCUCUCCUCAG
hsa-miR-143-3p	YP00205992	UGAGAUGAAGCACUGUAGCUC
hsa-miR-148a-3p	YP00205867	UCAGUGCACUACAGAACUUUGU
hsa-miR-151a-3p	YP00204576	CUAGACUGAAGCUCCUUGAGG
hsa-miR-151a-5p	YP00204007	UCGAGGAGCUCACAGUCUAGU
hsa-miR-16-2-3p	YP00204309	CCAAUAUUACUGUGCUGCUUUA
hsa-miR-16-5p	YP00205702	UAGCAGCACGUAAAUAUUGGCG
hsa-miR-181a-5p	YP00206081	AACAUUCAACGCUGUCGGUGAGU
hsa-miR-191-5p	YP00204306	CAACGGAAUCCCAAAAGCAGCUG
hsa-miR-199a-5p	YP00204494	CCCAGUGUUCAGACUACCUGUUC
hsa-miR-19b-3p	YP00204450	UGUGCAAAUCCAUGCAAAACUGA
hsa-miR-215-5p	YP00204598	AUGACCUAUGAAUUGACAGAC
hsa-miR-22-5p	YP00204255	AGUUCUUCAGUGGCAAGCUUUA
hsa-miR-223-3p	YP00205986	UGUCAGUUUGUCAAAUACCCCA
hsa-miR-29a-3p	YP00204698	UAGCACCAUCUGAAAUCGGUUA
hsa-miR-29b-3p	YP00204679	UAGCACCAUUUGAAAUCAGUGUU
hsa-miR-30e-5p	YP00204714	UGUAAACAUCCUUGACUGGAAG
hsa-miR-326	YP00204512	CCUCUGGGCCCUUCCUCCAG
hsa-miR-328-3p	YP00204364	CUGGCCCUCUCUGCCCUUCCGU
hsa-miR-33a-5p	YP00205690	GUGCAUUGUAGUUGCAUUGCA
hsa-miR-376a-3p	YP00204508	AUCAUAGAGGAAAAUCCACGU
hsa-miR-376c-3p	YP00204442	AACAUAGAGGAAAUUCCACGU
hsa-miR-409-3p	YP00204358	GAAUGUUGCUCGGUGAACCCCU
hsa-miR-424-5p	YP00204736	CAGCAGCAAUUCAUGUUUUGAA
hsa-miR-502-3p	YP00204043	AAUGCACCUGGGCAAGGAUUCA
hsa-miR-627-5p	YP00205979	GUGAGUCUCUAAGAAAAGAGGA
hsa-miR-744-5p	YP00204663	UGCGGGGCUAGGGCUAACAGCA
hsa-miR-766-3p	YP00204499	ACUCCAGCCCCACAGCCUCAGC
